# Acute Hepatitis and Invasive Bacteremia Caused by Aeromonas caviae in an Immunocompetent Adult: A Case Report and Literature Review

**DOI:** 10.7759/cureus.96200

**Published:** 2025-11-06

**Authors:** Ana Coimbra, Joana Duarte, André Carmo, Fabiana Pimentel, Jandira Lima

**Affiliations:** 1 Internal Medicine, Unidade Local de Saúde de Coimbra, Coimbra, PRT

**Keywords:** 80 and over, aeromonas caviae, aged, bacteraemia, case reports, gram-negative bacterial infections, hepatitis, immunocompetence, urban population

## Abstract

*Aeromonas* spp. are emerging pathogens with recognized capacity to cause invasive infections, especially in immunocompromised hosts or in the context of environmental exposure to contaminated water. However, their clinical relevance in urban-dwelling, immunocompetent patients remains underappreciated, partly due to diagnostic challenges and lack of routine microbiological screening. We report the case of an 83-year-old immunocompetent woman, living in an urban setting, who presented with fever and altered mental status. Laboratory investigations revealed acute hepatitis with markedly elevated liver enzymes and inflammatory markers. Blood cultures identified *Aeromonas caviae*, resistant to carbapenems and susceptible to third-generation cephalosporins. The patient recovered with appropriate antibiotic therapy. Stool cultures were not obtained, as no gastrointestinal symptoms were present. This case highlights the potential of *A. caviae* to cause extraintestinal invasive disease with hepatic involvement in immunocompetent, urban-dwelling individuals. It underscores the importance of maintaining a high index of suspicion for *Aeromonas* spp. in febrile syndromes with liver involvement, regardless of gastrointestinal symptoms or classical exposure history. Early identification requires specific microbiological testing, which may not be routinely performed. Clinicians should consider *Aeromonas* spp. in the differential diagnosis of acute hepatitis and fever, even in urban settings without known environmental exposure. Failure to identify this pathogen may delay appropriate therapy and compromise outcomes. Broader awareness and targeted diagnostic strategies are warranted to improve clinical care and public health surveillance.

## Introduction

The genus *Aeromonas -* particularly species such as *A. hydrophila*, *A. veronii*, and *A. caviae - *has increasingly been recognized as a human pathogen responsible for a wide spectrum of clinical syndromes, ranging from mild gastroenteritis to severe systemic infections [1-4]. While bacteremia caused by *Aeromonas* spp. has been well documented in immunocompromised individuals or those with hepatobiliary disease, malignancy, or haematologic disorders [1,2], its occurrence in immunocompetent patients remains exceedingly rare and poorly characterized [5-7].

*Aeromonas caviae*, in particular, is more frequently associated with pediatric and opportunistic infections, and its isolation in blood cultures of immunocompetent individuals is uncommon. Even more unusual is the development of significant hepatobiliary involvement, such as acute hepatitis, in the absence of known liver disease or biliary tract pathology. In fact, although *A. caviae* has been implicated in some extraintestinal presentations, the literature contains only a few descriptions of clinically significant hepatic dysfunction linked to *Aeromonas* species, and usually in the context of polymicrobial or nosocomial settings [5].

Compounding the rarity of this clinical presentation, most reported cases of *Aeromonas* bacteremia typically follow gastrointestinal manifestations such as diarrhea or vomiting, particularly after exposure to freshwater or ingestion of contaminated food or water. The absence of gastrointestinal prodromes, as in the current case, adds a further layer of diagnostic challenge and highlights the atypical nature of this presentation [8].

This report describes a rare case of *A.* *caviae* bacteremia associated with acute hepatitis in an 83-year-old, immunocompetent, urban-dwelling woman with no identifiable predisposing conditions. To our knowledge, this constellation of findings - isolated bloodstream infection by *A. caviae*, marked liver injury without underlying disease, and no gastrointestinal symptoms - has not been previously documented in the literature. This case underscores the need to broaden clinical suspicion for *Aeromonas* infections in unusual presentations and contributes novel insight to the evolving spectrum of disease caused by this genus.

## Case presentation

An 83-year-old woman presented to the emergency department (ED) of a tertiary care hospital in central Portugal with acute altered mental status, generalized weakness, and fever. Her family reported a three-day history of confusion and reduced oral intake, with marked deterioration from baseline function in the preceding week, culminating in a febrile episode and decreased responsiveness on the day of admission.

The patient lived in an urban setting with family support and complete functional independence prior to presentation. Her past medical history included well-controlled hypertension, dyslipidemia, and type II diabetes. She denied any prior liver disease, malignancy, autoimmune condition, immunosuppressive therapy, or recent antibiotic use. The patient was fully vaccinated against COVID-19 and influenza, without recent travel, exposure to untreated water sources, or contact with animals.

The patient’s chronic medications included metformin 850 mg twice daily, carbamazepine 200 mg AM and 400 mg PM, atorvastatin 20 mg daily, irbesartan 300 mg every morning, amlodipine 10 mg with lunch, bisoprolol 2.5 mg in the evening, sertraline 50 mg at dinner, pantoprazole 20 mg daily, and bioflavonoids 500 mg daily. All medications had been taken chronically with no recent additions, dosage changes, or use of over-the-counter drugs, supplements, or known hepatotoxic agents prior to symptom onset. The patient reported a remote allergy to penicillin, though without a clearly documented reaction.

Upon arrival, her vital signs were notable for a temperature of 37.8°C, blood pressure of 156/68 mmHg, heart rate 75 bpm, respiratory rate of 18/minute, and oxygen saturation of 96% on ambient air. Physical examination revealed no signs of abdominal pain, jaundice, hepatosplenomegaly, or focal neurological deficits. The patient was disoriented to time and place but able to follow simple commands. There were no signs of meningeal irritation or rash. Cardiac, pulmonary, and abdominal examinations were unremarkable.

Initial laboratory evaluation revealed a significant inflammatory response, with marked hepatocellular injury: aspartate aminotransferase (AST) greater than 30 times the upper limit of normal and alanine aminotransferase (ALT) approximately 20 times the upper limit of normal. Alkaline phosphatase (ALK) and gamma-glutamyl transferase (GGT) were also elevated, along with elevated lactate dehydrogenase, while total bilirubin was only slightly elevated. Renal function was preserved, and coagulation parameters were within reference ranges. Serum albumin was normal, and lactate was not elevated. Urinalysis was unremarkable. Two sets of blood cultures were collected. Initial laboratory findings are summarized in Table 1.

**Table 1:** Initial laboratory findings on admission. CRP: C-reactive protein; WBC: white blood cells; AST: aspartate aminotransferase; ALT: alanine aminotransferase; ALP: alkaline phosphatase; GGT: gamma-glutamyl transferase; LDH: lactate dehydrogenase; INR: international normalized ratio

Test	Result	Reference Range	Comment
CRP	23.9 mg/dL	<0.5 mg/dL	Markedly elevated
WBC	11.0 × 10^9^/L	3.6-10.5 × 10^9^/L	Mild leukocytosis
AST	1038 U/L	<31 U/L	Severe cytolysis
ALT	758 U/L	<34 U/L	Severe cytolysis
ALP	300 U/L	30-120 U/L	Elevated
GGT	449 U/L	<38 U/L	Markedly elevated
LDH	1075 U/L	<247 U/L	Elevated
Total bilirubin	2.0 mg/dL	<1.2 mg/dL	Slightly elevated
Direct bilirubin	1.4 mg/dL	<0.5 mg/dL	Elevated
INR	1.07	~0.8-1.2	Normal
Albumin	3.7 g/dL	3.5-5.2 g/dL	Normal
Lactate	Normal	<2.0 mmol/L (lab dependent)	Not elevated
Urinalysis	Unremarkable	-	No abnormalities

**Figure 1:**
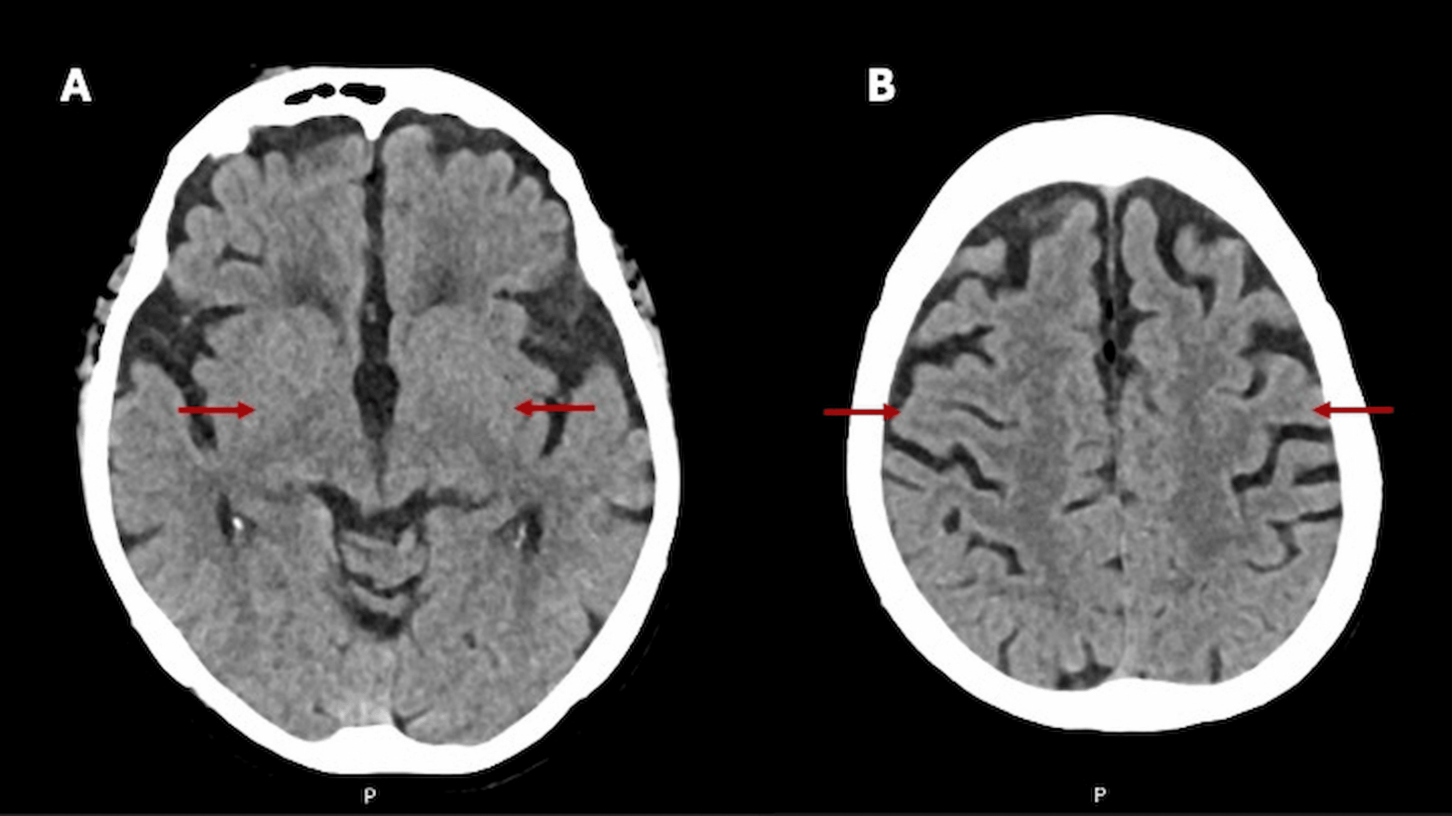
Axial non-contrast cerebral CT images revealing no acute pathology. (A) Cut at the level of the basal ganglia shows preserved symmetry, no hemorrhage, and no focal lesions. The arrow indicates the basal ganglia region, where no ischemic or space-occupying changes were detected. (B) Cut at the high convexity level demonstrates diffuse sulcal prominence, consistent with age-related cortical atrophy. No mass effect or midline shift is seen.

A non-contrast cerebral CT scan was performed to rule out acute structural causes for the altered mental status. No evidence of hemorrhage, infarction, mass effect, or midline shift was found. Chronic microvascular ischemic changes and age-appropriate cortical atrophy were noted, with well-preserved CSF spaces (Figure 1).

**Figure 2:**
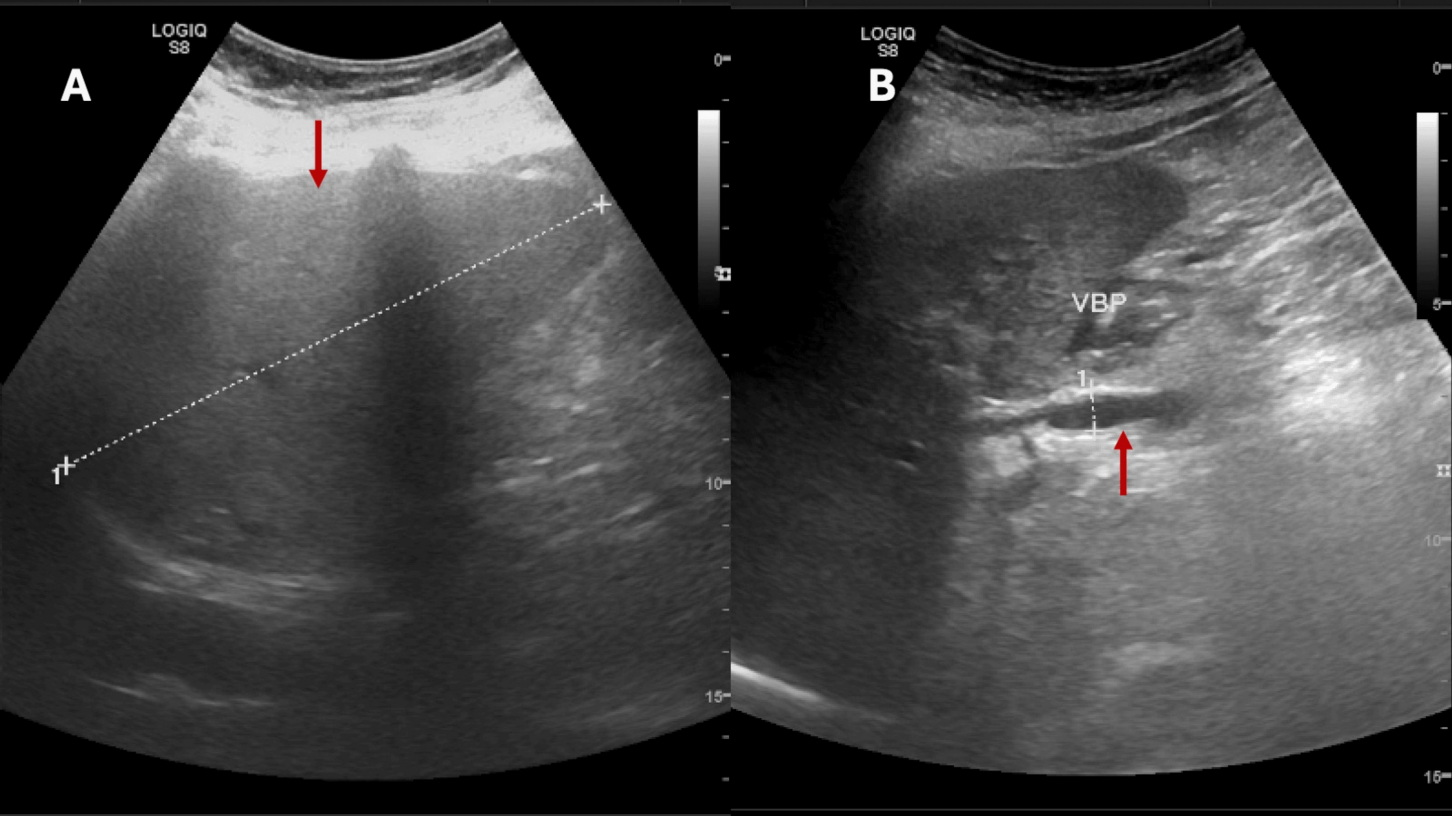
Abdominal ultrasound images obtained during the diagnostic workup. (A) Longitudinal view of the liver showing a slightly heterogeneous but globally preserved echotexture, with no focal lesions or perihepatic fluid (arrow). Findings were interpreted as suggestive of diffuse steatosis. (B) View of the extrahepatic biliary tract showing a mildly dilated common bile duct, measuring approximately 9 mm (arrow), without intrahepatic biliary dilation.

Abdominal ultrasonography was performed as part of the diagnostic evaluation. The liver appeared slightly heterogeneous and hyperechoic, suggestive of diffuse steatosis, with normal dimensions and no focal lesions. The common bile duct was mildly dilated (9 mm) but without associated intrahepatic biliary dilation. No ascites was detected (Figure 2).

Given the altered mental status and elevated liver enzymes in the absence of identifiable hepatobiliary or structural central nervous system pathology, a working diagnosis of acute hepatitis of unclear etiology with systemic inflammatory response was established. Empirical treatment with intravenous ceftriaxone (2g daily) and supportive care was initiated, and she was admitted to the internal medicine ward for inpatient management and further investigation.

The following day, both sets of blood cultures grew *A. caviae*, resistant to amoxicillin-clavulanate but sensitive to third-generation cephalosporins and fluoroquinolones. A comprehensive workup was undertaken to investigate the cause of acute liver injury. Viral serologies, including hepatitis A, B, C, and E, HIV, and cytomegalovirus (CMV)/Epstein-Barr virus (EBV), were negative. There was no history of hepatotoxic medication use or recent alcohol intake. Autoimmune markers were not suggestive of autoimmune hepatitis, and no underlying liver disease was identified on a repeat ultrasound. The pattern of transaminase elevation, elevated inflammatory markers, and temporal correlation with bacteremia by *A. caviae* was consistent with an acute infectious hepatitis of bacterial origin.

A final diagnosis of *A. caviae* bacteremia associated with acute hepatitis was made, and empirical therapy was switched to levofloxacin following identification of the isolate and its susceptibility profile.

Further laboratory investigation showed a gradual decline in hepatic enzyme levels over the ensuing days. Stool cultures were not obtained, as the patient remained asymptomatic from a gastrointestinal standpoint throughout hospitalization, with no episodes of diarrhea, nausea, or vomiting.

The patient’s clinical condition improved steadily. By day 5, she was afebrile, alert, and mentally lucid, and tolerating an oral diet. Liver enzymes continued to trend downward, and inflammatory markers returned to baseline. Antibiotic therapy was maintained for a total of 14 days. Table 2 summarizes the progressive improvement in hepatic and inflammatory markers over the course of hospitalization, following initiation of targeted antibiotic therapy.

**Table 2:** Temporal evolution of key laboratory parameters following initiation of antibiotic therapy. Laboratory trends are presented across four time points: Day 0 (start of antibiotics), Day 3, Day 9, and Day 15 of hospitalization. LDH: lactate dehydrogenase; AST: aspartate aminotransferase; ALT: alanine aminotransferase; ALK: alkaline phosphatase; GGT: gamma-glutamyl transferase; CRP: C-reactive protein; T Brb: total bilirrubin; D Brb: direct bilirrubin

Test	Day 0	Day 3	Day 9	Day 15	Reference Range
LDH (U/L)	1075.0	174.0	199.0	241.0	<247
AST (U/L)	1038.0	143.0	38.0	23.0	<31
ALT (U/L)	758.0	324.0	71.0	26.0	<34
ALP (U/L)	300.0	199.0	137.0	90.0	30-120
GGT (U/L)	449.0	304.0	198.0	98.0	<38
T Brb (mg/dL)	2.0	1.4	0.5	0.4	<1.2
D Brb (mg/dL)	1.4	1.0	0.9	0.9	<0.5
CRP (mg/dL)	23.9	14.0	1.04	0.5	<0.5

She was discharged home in stable condition on day 15 of hospitalization, with instructions for outpatient follow-up. At a two-week follow-up visit, she reported full return to baseline function, with complete resolution of symptoms and normal laboratory values.

## Discussion

The genus *Aeromonas* comprises facultative anaerobic Gram-negative bacilli commonly found in aquatic environments [4]. Among them, *A.* caviae is primarily associated with gastroenteritis, while invasive infections such as bacteremia and hepatobiliary involvement remain uncommon and are typically reported in immunocompromised hosts.

In this case, the diagnosis of acute hepatitis and bacteremia due to *A. caviae* in an elderly, immunocompetent, urban-dwelling woman without prior hepatobiliary disease, autoimmune conditions, or malignancy underscores a rare and clinically relevant manifestation of an emerging pathogen [1]. While liver involvement has been occasionally described in *Aeromonas* infections, it typically occurs in the context of cirrhosis, malignancy, or post-liver transplantation [9]. In our patient, all standard hepatic risk factors were absent, and the acute hepatitis resolved with targeted antimicrobial therapy, implicating a direct infectious cause. To our knowledge, this represents one of the few documented instances of *A. caviae *hepatitis in such a context.

Moreover, the patient exhibited no gastrointestinal symptoms throughout the course of illness, making the diagnosis less intuitive. Several case series emphasize that while* A. caviae* can be isolated from stool, asymptomatic or extraintestinal presentations are underrecognized, particularly in bloodstream infections, and are mostly described in immunocompromised populations [7]. Importantly, retrospective analyses have demonstrated that *A. caviae* tends to be less virulent than *A. hydrophila* but can still cause serious infections, especially in elderly individuals with comorbidities.

From a diagnostic standpoint, this case highlights the importance of considering *Aeromonas* species in blood cultures, even in patients without classic risk profiles or gastrointestinal symptoms. Identification was made possible by modern blood culture systems and species-level classification, emphasizing the need for microbiology laboratories to accurately characterize *Aeromonas* isolates due to their variable antibiotic susceptibilities [1]. *A. caviae *strains are generally susceptible to fluoroquinolones, aminoglycosides, and third-generation cephalosporins, although resistance patterns vary geographically and over time [3,5,10]. Previous reports of* A. caviae *bacteremia, particularly in cancer patients, have documented variable susceptibility profiles and resistance to carbapenems, underscoring the importance of tailored antimicrobial therapy [9].

Although *Aeromonas* bacteremia is increasingly recognized [6], its role as a hepatotropic pathogen remains underexplored [3,4]. The mechanisms of hepatic injury in such infections are speculative but may involve direct bacterial invasion, endotoxin release, or immune-mediated hepatocellular damage [11]. Elevated transaminases, cholestatic enzymes, and systemic inflammatory markers, as observed in our patient, lend support to this theory. *A. caviae*, although generally considered less virulent than *A. hydrophila*, expresses several virulence factors - including enterotoxins, hemolysins, and type III secretion systems - that may underlie its capacity for extraintestinal invasion and hepatic involvement [2].

While the exact source of infection in this case remains uncertain, possible urban exposures such as municipal water, inadequately washed produce, or contaminated kitchen environments should be considered.

A limitation of this report is the absence of a liver biopsy for confirmation. However, the acute and reversible hepatic enzyme elevations in temporal association with bacteremia, in the absence of alternative explanations, strongly support the infectious etiology. Additionally, stool cultures were not performed, which might have offered insight into intestinal colonization.

## Conclusions

This case broadens the clinical spectrum of *A. caviae* infections, illustrating that severe hepatic involvement and bacteremia can occur in immunocompetent hosts, even in the absence of gastrointestinal symptoms or predisposing conditions. It underscores the importance of early microbiological investigation and species identification, which enabled appropriate antibiotic therapy and a favorable clinical outcome.

While this report illustrates a rare manifestation, its findings support the growing recognition of *A. caviae* as an emerging extraintestinal pathogen, particularly in elderly patients. Further studies are needed to better understand its pathogenesis, optimize therapeutic approaches, and identify patient populations at risk for severe disease.
